# Donkey Gelatin and Keratin Nanofibers Loaded with Antioxidant Agents for Wound Healing Dressings

**DOI:** 10.3390/gels10060391

**Published:** 2024-06-08

**Authors:** Maria Râpă, Carmen Gaidau, Laura Mihaela Stefan, Andrada Lazea-Stoyanova, Mariana Daniela Berechet, Andreea Iosageanu, Ecaterina Matei, Virginija Jankauskaitė, Cristian Predescu, Virgilijus Valeika, Aistė Balčiūnaitienė, Snezana Cupara

**Affiliations:** 1 Faculty of Material Science and Engineering, National University of Science and Technology Politehnica Bucharest, 060042 Bucharest, Romania; maria.rapa@upb.ro (M.R.); ecaterina.matei@upb.ro (E.M.); cristian.predescu@upb.ro (C.P.); 2 The National Research & Development Institute for Textiles and Leather-Division Leather and Footwear Research Institute, 031251 Bucharest, Romania; marianadanielaberechet@yahoo.co.uk; 3 Department of Cellular and Molecular Biology, National Institute of Research and Development for Biological Sciences, 060031 Bucharest, Romania; laura.stefan@incdsb.ro (L.M.S.); andreea.iosageanu@incdsb.ro (A.I.); 4 Low Temperature Plasma Department, National Institute for Laser, Plasma and Radiation Physics, 409 Atomistilor Street, 077125 Magurele, Romania; andrada@infim.ro; 5 Faculty of Mechanical Engineering and Design, Kaunas University of Technology, 51424 Kaunas, Lithuania; virginija.jankauskaite@ktu.lt; 6 Faculty of Chemical Technology, Kaunas University of Technology, Radvilenu pl. 19, 50254 Kaunas, Lithuania; virgilijus.valeika@ktu.lt; 7 Lithuanian Research Centre for Agriculture and Forestry, Institute of Horticulture, 54333 Babtai, Lithuania; aiste.balciunaitiene@lammc.lt; 8 Faculty of Medical Sciences, University of Kragujevac, Svetozara Markovića 69, 34000 Kragujevac, Serbia; snezanacupara@yahoo.com

**Keywords:** gelatin, keratin, nanofibers, donkey hide, natural antioxidants, wound healing

## Abstract

Acute and chronic wounds present a significant healthcare challenge, requiring innovative solutions for effective treatment. The exploitation of natural by-products with advanced cell regeneration potential and plant-based materials, which possess bioactive properties, is an innovative topic in wound management. This study investigates the potential of donkey gelatin and keratin for blending with natural bioactive extracts such as sumac, curcumin, and oak acorn to fabricate antioxidant and antimicrobial nanofibers with accelerated wound healing processes. The fabricated nanofibers possess good in vitro biocompatibility, except for the sumac-based donkey nanofibers, where cell viability significantly dropped to 56.25% (*p* < 0.05 compared to non-treated cells). The nanofiber dimensions showed structural similarities to human extracellular matrix components, providing an ideal microenvironment for tissue regeneration. The donkey nanofiber-based sumac and curcumin extracts presented a higher dissolution in the first 10 min (74% and 72%). Curcumin extract showed similar antimicrobial and antifungal performances to rivanol, while acorn and sumac extracts demonstrated similar values to each other. In vitro tests performed on murine fibroblast cells demonstrated high migration rates of 89% and 85% after 24 h in the case of acorn and curcumin nanofibers, respectively, underscoring the potential of these nanofibers as versatile platforms for advanced wound care applications.

## 1. Introduction

The increasing prevalence of diseases caused by blows, traffic accidents, cuts, scalds, and burns (chronic diseases) and the aging population bring into focus the considerable burden and care of wounds. The success rate of wound healing outcomes entails the following steps: fast hemostasis, adequate inflammation, mesenchymal cell differentiation, growth and migration to the site of injury, formation of new blood vessels, rapid re-epithelialization and appropriate synthesis, proper cross-linking, and orientation of collagen to strengthen the healing tissue [1]. The ineffectiveness of current antibiotics in infections with resistant pathogens (multi-drug-resistant bacteria) constitutes the greatest threat to global health [2,3,4]. The World Health Organization (WHO) highlighted that the inefficiency of antibiotics used to treat the bacterial infection may result in the deaths of almost 10 million people annually by 2050 [5] as well as prolong the patient’s hospital stay.

Modern dressings are created from multifunctional materials with the aim of improving the rate of wound recovery by speeding up the healing process, offering physical and antimicrobial protection, and maintaining the moisture balance of the wound microclimate [6]. The restoration of dermal tissue through the exploitation of natural by-products with advanced cell regeneration potential, compared to the existing products on the market, is an innovative topic in wound management. Collagen, a biopolymer known for its regenerative and tissue reconstruction properties, has been extensively investigated for the design of natural wound dressings. Collagen, found in bones, muscles, skin, and tendons, is produced by fibroblasts. Biomaterials containing collagen promote certain cells, such as macrophages and fibroblasts, thereby improving wound healing [7]. Keratin is a group of proteins that form cysteine-rich filaments, constituting the main component of hair, hooves, wool, nails, horns, and feathers [8]. The roles of keratins are to encourage the growth of keratinocytes and maintain epithelial integrity within medical dressing materials [9].

Donkey hide (*Equus asinus* L.) is a basic raw material for the preparation of a gelatin (*Colla corii asini*) used as a food and drug to treat anemia in traditional Chinese medicine for over 2000 years [10]. More than 58 compounds were isolated from donkey gelatin, among which amino acids (hydroxyproline, the fingerprint amino acid for collagen), proteins (collagen α1 (I), collagen α2 (I), and albumin), polysaccharides (dermatan sulfate), volatiles, and inorganic substances (calcium oxide and sodium oxide) [10]. It was reported that the low-molecular weight peptides obtained from the gelatin hydrolysates of donkey hide are responsible for their high antioxidant properties [11]. These peptides are an effective anti-photoaging agent against UVB radiation and increase the synthesis of type I procollagen [12].

In the management of innovative wound healing, the antioxidant and antimicrobial dressings can prevent the wound infection and promote wound healing through the active release of antimicrobial agents or passively by their antiseptic surfaces [13,14]. For instance, curcumin (diferuloylmethane), a naturally derived polyphenol found in turmeric root, demonstrated anti-inflammatory and antioxidant characteristics, promoting keratinocyte migration and proliferation and showing potential benefits during the maturation phase of wound treatment [15,16,17,18]. To overcome the challenges associated with the low solubility of curcumin in aqueous solutions and limited bioavailability, researchers have investigated its incorporation into different carriers, such as chitosan/hyaluronic acid (HA)/poly(vinyl alcohol) (PVA)-magnetic montmorillonite (mMMt2) [15] and PCL-chitosan [19]. These prepared nanofibers demonstrated effectiveness in inhibiting the growth of *E. coli* and *S. aureus* bacteria [15] and had a positive impact on the viability and proliferation of human dermal fibroblasts (HDFs) [19]. Additionally, nanofibers containing curcumin, γ-polyglutamic acid, and gum arabic exhibited therapeutic potential in wound healing by accelerating the re-epithelialization process, enhancing wound contraction, and promoting the regeneration of new blood vessels and hair follicles [20]. Literature data highlight the use of sumac fruits (*Rhus coriaria* L.) and acorns (*Quercus brantii* Lindl.) in various domains such as industry, pharmaceuticals, and nutrition [21,22,23]. At 5 mg mL^−1^ and 10 mg mL^−1^ concentrations of sumac extracted from fruits, it was found to accelerate the healing of experimentally induced wounds in male Wistar rats [24]. Acorns are rich in polyphenols like gallic and ellagic acids [25], depending on the oak species, and display anti-inflammatory, antibacterial, hypoglycemic, or antifungal activities [26]. One paper reported the use of acorn extract in contents of 0.5%, 1%, and 1.5% (*w*/*v*) as a natural cross-linker and antibacterial agent for chitosan/gelatin/poly(vinyl alcohol)(PVA) nanofibers for wound-healing applications [23]. In this context, our research strategy involved the use of plant-derived extracts, such as curcumin, acorn, and sumac, by incorporating them into a mixture of gelatin and keratin extracted from donkey hide for developing antimicrobial and antioxidant nanofibers with potential application in wound healing.

Electrospinning is a relatively straight-forward procedure capable of rapidly producing nanofibrous structures with a high surface area-to-volume ratio and tunable fiber properties [27]. Nanofibers for innovative medical dressings manufactured using the electrospinning technique must fulfill several requirements: to absorb excess exudates, provide and maintain a moist environment or an adequate water vapor transmission rate, possess smaller pores compared to fibers produced using traditional methods, exhibit good cellular adhesion to support cell proliferation, and enhance the healing process. Nonetheless, there is evidence that the use of inflammable liquids with high shear strength and voltage can potentially generate permanent denaturation of the collagen fibrous structure [28]. Our previous publications reported the fabrication of nanofibrous wound dressings by the electrospinning process of different protein extracts, such as collagen derived from cattle hides [29,30], rabbit skins [31,32], fish scales [33,34], donkey hides [34], or keratin extracted from sheep wool [35], loaded with various non-active antimicrobial agents and having advanced regenerative properties for acute and chronic wound healing.

The aim of this paper is to combine the gelatin and keratin extracted from donkey hide with natural bioactive extracts such as curcumin, sumac, and acorn to obtain nanofibers with a potential application in accelerating the healing process of dermal wounds, leveraging their anti-inflammatory, antioxidant, and antimicrobial properties. Our hypothesis was that the use of bioactive nanofibers with small fiber diameters and fine pores would quickly prevent bacteria penetration into the wound area and stimulate cell proliferation and skin regeneration. This study presents a novel strategy for the fabrication of bioactive nanofibers, expanding the potential uses of readily available natural resources such as curcumin, acorn, and sumac, as well as valuing animal-derived by-products like donkey hide and hair, contributing to the advancement of Sustainable Development Goals (SDGs) set by the United Nations for achievement by 2030.

## 2. Results and Discussion

### 2.1. Physical-Cheminal Properties of Gelatin and Gelatin/Keratin Loaded with Bioactive Agents

The extraction yields of gelatin and keratin from the donkey hide and hair, were 30 ± 5% and 80 ± 10%, respectively, according to raw material weight.

Table 1 displays the main properties of gelatin extracted from donkey hide and gelatin mixed with keratin hydrolysate extracted from donkey hair.

Gel strength of the gelatin is the main parameter for assessing the quality of the gelatin [36], being induced by the attraction between hydrogen bonds from water and the carboxyl ends of the amino acids [37]. These interactions lead to the formation of more aggregate macromolecules. The composition of the amino acid residues in gelatin consists of glycine, proline, and hydroxyproline, which are connected together through peptide bonds [38]. Hydrogen bonds between the inter-amino acid residues ensure the triple-helix structure of gelatin, providing strength and stability to the gelatin network. Measuring the gel strength of gelatin is vital for both control purposes and determining the amount of gelatin needed for a specific application [39]. Donkey hide gelatin is traded as a Chinese traditional remedy and has 250 g of gel strength [40]. Gelatins of 225–325 g gel strength are high-bloom gelatins used for preparing desserts, meat-based food, soft capsules, and ballistic items [41]. As compared to the literature data showing the gel strengths of calf skin gelatin and pork skin gelatin of 336.87 g and 308.07 g, respectively [42], our donkey hide gelatin is a high bloom gelatin with superior values. The combination of donkey gelatin with keratin has a higher bloom test value due to the higher pH value, which influences molecular associations between collagen and keratin peptides compared to donkey gelatin alone [43].

The physical-chemical characteristics of bioactive formulations before the electrospinning process are displayed in Table 2.

The high electrical conductivity for DKGS, DKGA, and DKGC nanofibers is due to the presence of minerals, vitamins, and unsaturated fatty acids in the composition of sumac [21,44,45], acorn [21,46], and curcumin [47], as well as donkey hide [10]. DGK, DGKC, and DGKR solutions exhibited a pH around 9 that was explained by the formation of OH− ions with a buffering effect. The pH value of around 7 for DKGS and DKGA could be explained by the acidic pH reported for sumac fruit extracted in an aqueous solution [48] and the total fatty acid found in acorn products [46]. Salinity, expressed by the dissolved salts in the solutions of DKG loaded with bioactive extracts, was directly related to the electrical conductivity values. The ionic compositions of bioactive formulations rich in Ca, Mg, and Cl ions (Table 3) led to increased salinity values compared with those for DKG. Rheological data (Table 2) showed an increase in the viscosity and shear stress of DKG solutions loaded with bioactive extracts compared with the original solution. The physical-chemical parameters for the investigated donkey gelatin-keratin loaded with different plant extracts depend on the geographical and environmental conditions where the plants were collected.

**Figure 1:**
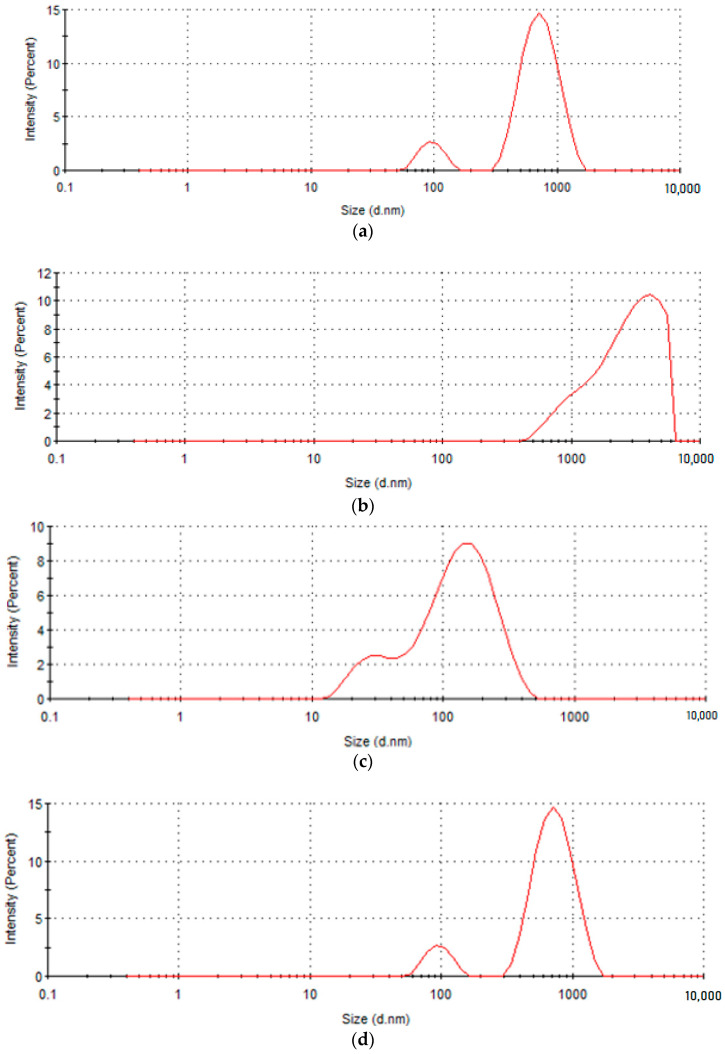
Particle diameter distributions of (**a**) donkey keratin, (**b**) donkey keratin-gelatin, (**c**) donkey keratin-gelatin mixed with curcumin extract, and (**d**) donkey keratin-gelatin mixed with acorn extract were assessed using the Dynamic Laser Scattering (DLS) technique.

Figure 1 shows the size distribution of particles measured for the solutions prepared for the electrospinning process after the centrifugation step.

Large polydispersity indices (Pdi) for keratin of 0.558, curcumin of 0.477, and acorn extracts of 0.399 indicate a very broad size distribution, overlapping with that of DKG (0.553). For DKGA and DKGC, three distinct peaks were observed, suggesting a potential cross-linking of gelatin and keratin, resulting in the generation of larger molecules. This behavior is related to the increased viscosity as the bioactive agents were loaded into DKG (Table 2). For DKGA, the peak diameters were recorded in the range of 20.79 ± 6.654 nm to 157.1 ± 74.6 nm. The small peak in intensity occurring at 5386 nm could be due to the sample preparation. For DKGC, the main two peaks with diameters of 29.87 ± 80.14 nm and 155.7 ± 80.14 nm, respectively, were observed. The smallest peak diameter size was encountered for DKGA (20.79 ± 6.654 nm), while the highest peak diameter size of 2951 ± 1460 nm was detected by DKG. Z-average shows values of 1942 nm for DKG, 119.5 nm for DKGA, and 78.69 nm for DKGC solutions. Zeta potential indicated negative values due to the abundance of anionic amino acid residues [37]. A slow increase in the stability of particles for DKG loaded with bioactive extracts was recorded around −16.5 mV, compared with DKG and DKGR, for which the zeta potential was −15.9 ± 3.19 mV and −13.1 ± 3.44 mV, respectively.

### 2.2. SEM/EDS Analysis

**Figure 2:**
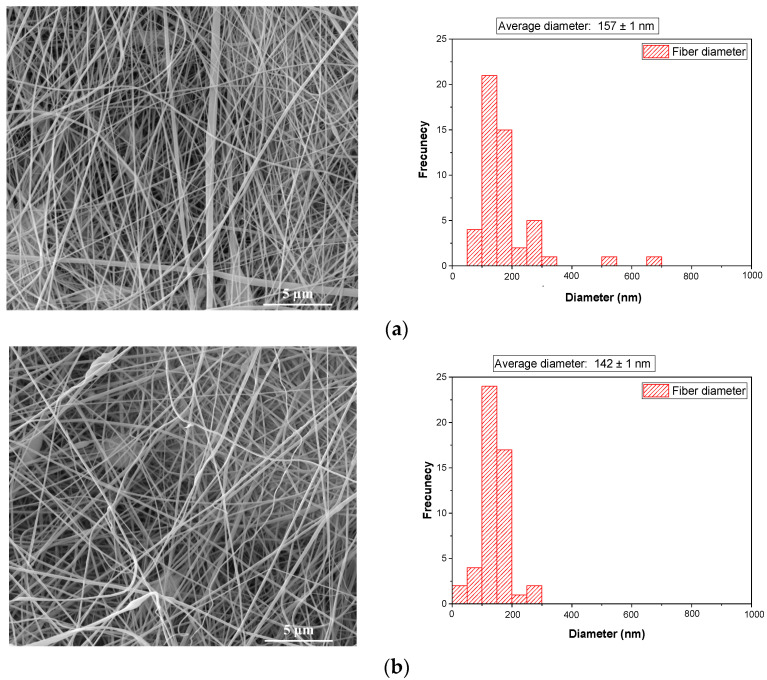

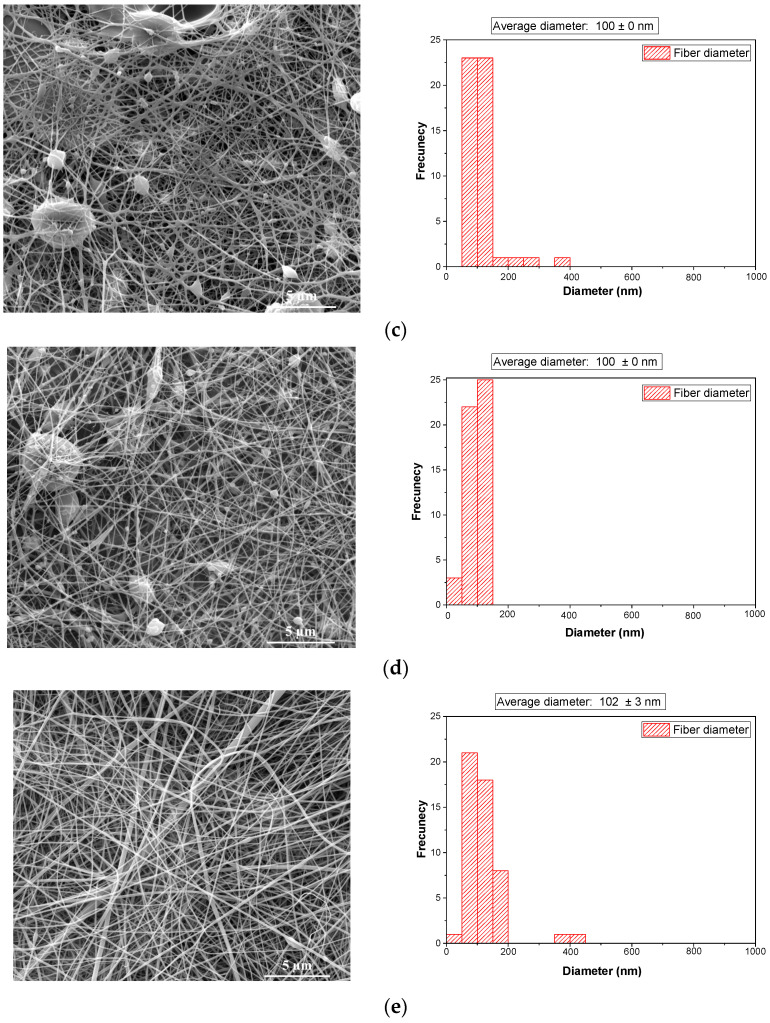
SEM images and average diameter of: (**a**) donkey gelatin with keratin (DKG) nanofibers; (**b**) donkey gelatin with keratin and sumac extract (DKGS) nanofibers; (**c**) donkey gelatin with keratin and curcumin extract (DGKC) nanofibers; (**d**) donkey gelatin with keratin and acorn extract (DKGA) nanofibers; (**e**) donkey gelatin with keratin and rivanol (DKGR) nanofibers, at magnifications of 10,000×.

The morphology and average diameter of bioactive nanofibers were examined via SEM (Figure 2a–e).

The morphology of bioactive nanofibers is influenced by the composition of the formulation, the physical-chemical properties of the solution, and the electrospinning parameters. As depicted in Figure 2, DKG and DKGS formulations show nanofibers without beads and defects, in contrast to the morphology of DKGC and DKGA nanofibers. The electrical conductivity values for DKG and DKGS ranged between 0.5 mS/cm and 9.45 mS/cm, suggesting the generation of a stable electrospinning jet. This is associated with nanofiber dimensions, ranging from 142 ± 1 nm to 157 ± 1 nm (Figure 2a,b). In the case of nanofibers based on curcumin and acorn extracts, beads were observed (Figure 2d,e). These formulations exhibited high electrical conductivity values, suggesting possible inter- and intramolecular interactions, which may lead to the formation of beads. Interesting: DKGA and DKGC nanofibers show the smallest size dimensions of nanofibers, around 101 nm, as well as the occurrence of beads. The increase in the electrical conductivity of solutions due to the side components in the extracts of bioactive compounds leads to a decrease in the diameter of bioactive nanofibers, attributed to jet elongation. Also, this observation can be explained by the increased viscosity of DKG loaded with bioactive extracts (Table 2). This behavior, when nanofiber diameter decreases with the increase in conductivity of the electrospinning solution, was also observed for wool keratin blended with polyvinyl alcohol (PVA) [49]. The decrease in nanofiber diameters is expected to positively affect cell adhesion and growth. Al-Sudani et al. [50] reported a similar trend, noting an increase from 405.2 ± 107.8 nm to 571.7 ± 171.8 nm in the fiber diameter of polymethyl-methacrylate (PMMA)/gelatin impregnated with a propolis content of 10% to 50%.

The results obtained from DLS and SEM analyses showed that there is a difference between the fiber diameters. This discordance in diameter sizes of nanofibers may result from the nanoparticles being in a compressed condition during the SEM investigation while they were swollen when DLS was conducted [51].

However, the fabricated bioactive nanofibers showed an average diameter very similar to the diameter of ECM collagen fibers found in the skin, typically ranging from 50 nm to 500 nm. In an extensive study examining the average diameter of gelatin nanofibers prepared by electrospinning gelatins derived from different sources—bovine, donkey, rabbit, and fish scale—it was observed that the donkey gelatin exhibited the smallest nanofiber diameter (73.15 nm ± 3.37 nm) [34]. The authors concluded that the origin of gelatin and optimized electrospinning conditions are essential for achieving nanofibers with dimensions closely resembling those of the extracellular matrix (ECM). Another study also reported a size dimension between 120 and 215 nm for nanofibers based on acorn/chitosan/gelatin [23].

**Figure 3:**
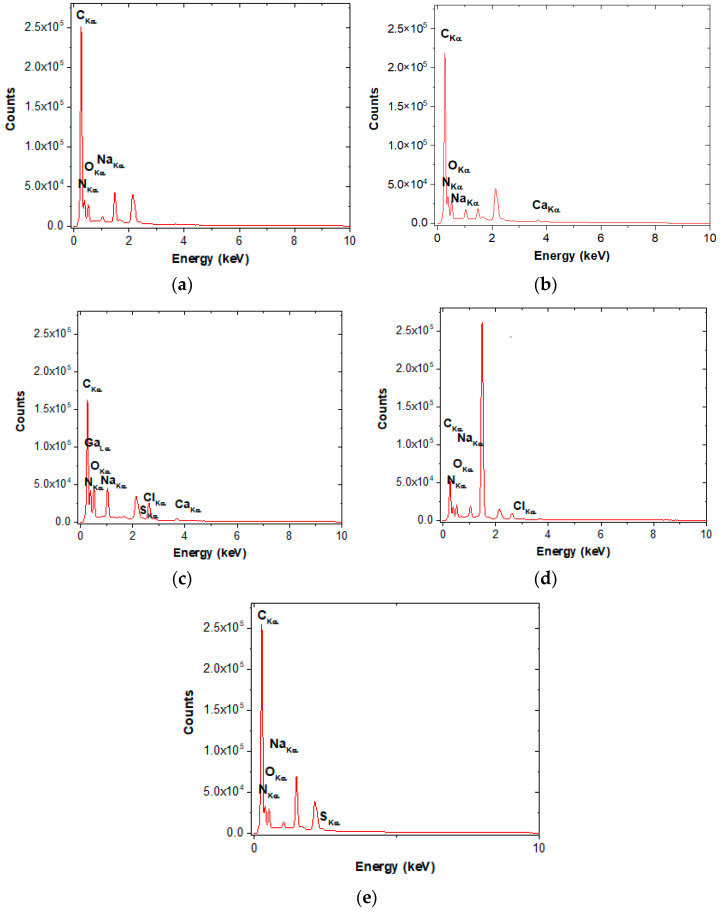
EDS patterns of bioactive nanofibers. (**a**) DKG, (**b**) DKGS, (**c**) DGKC, (**d**) DKGA, and (**e**) DKGR.

The chemical elements of electrospun nanofibers were determined using EDS analysis (Figure 3).

According to the data shown in Table 3, the main chemical elements found in DKG nanofibers are carbon, nitrogen, and oxygen. They appeared in all the fabricated nanofibers. In addition, calcium, aluminum, sulfur, and other trace elements appeared in the compositions of bioactive extracts. The gold presence originated from the processing of samples. Therefore, the peaks not related to the samples (Al_Kα_ and Au_M_ peaks) are not marked in the spectra. All DKG loaded bioactive extract nanofibers contained a decreased ratio of C/O compared with DKG nanofibers. This can be explained by the content of polyphenols in the composition of bioactive extracts.

### 2.3. ABTS Free Radical Cation Scavenging Assay

**Figure 4:**
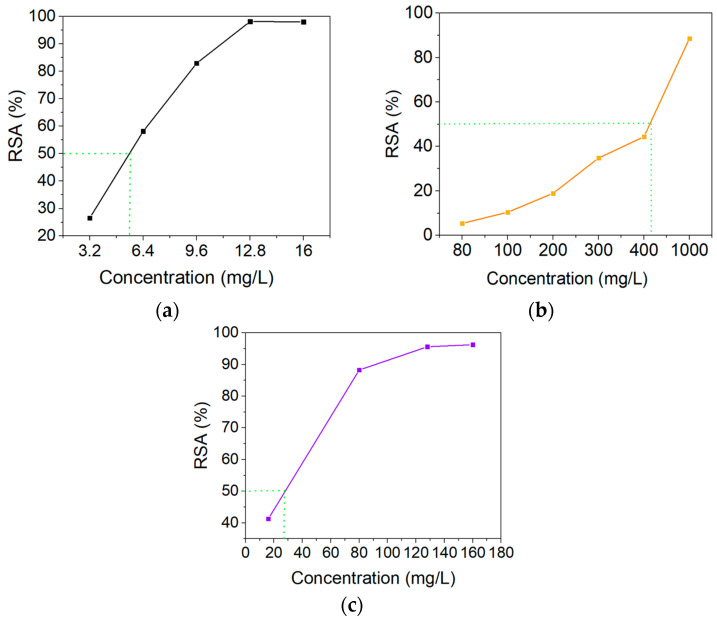
Half maximal inhibitory concentration (IC_50_) for (**a**) sumac, (**b**) curcumin, and (**c**) keratin extracts.

The free radical scavenging activity (IC_50_ values) of sumac, curcumin, and keratin extracts is shown in Figure 4.

The IC_50_ determined based on the ABTS^•+^ assay was 5.6 µg mL^−1^ for sumac extract, 475 µg mL^−1^ for curcumin extract, and 28 µg mL^−1^ for keratin. Acorn extracts from the fruits of *Quercus coccifera* L. (kermes oak) are frequently consumed as herbal coffee in some regions and have demonstrated high antioxidant activity (91.09 ± 1.71%) [52]. *Quercus cerris* seeds, another coffee substitute, were recognized for their high IC_50_ values of 271.61 µg mL^−1^ [53].

The results of the radical scavenging activity (*RSA*) assay of donkey gelatin-based nanofibers are presented in Table 4.

As was expected from the obtained IC_50_ data, the bioactive nanofibers showed high RSA values (Table 4). The antioxidant properties of keratin can be attributed to the cysteine amino acids, which can be converted into sulfoxide compounds through alkaline hydrolysis, as was previously reported [54]. Also, the results indicate that the polyphenolic compounds of sumac [55], curcumin [56], and acorn [57] are responsible for the most efficient antioxidant properties. The antioxidant activity of the acorn extracts by ABTS^•+^ assay was reported in the range of 17.20–35.21 µmol Trolox 6-hydroxy-2,5,7,8-tetramethylchroman-2-carboxylic acid equivalents (TE) per g of dry matter [58]. A similar high radical scavenging activity (*RSA*%) of 88.58 ± 0.15% was reported using a 2,2-diphenyl-1-picrylhydrazyl (DPPH) free radical scavenger for acorn shell extracted with ethanol [59].

### 2.4. Controlled Release of Sumac and Curcumin

**Figure 5:**
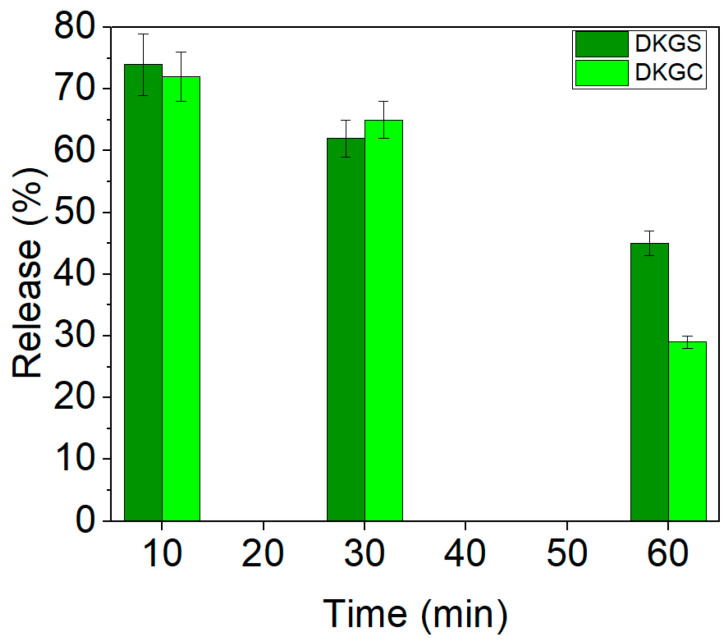
Release of sumac and curcumin extracts from the bioactive DKGS and DKGC nanofibers.

Figure 5 shows the release of curcumin and sumac extracts from DKG-based nanofibers.

The DKGS and DKGC nanofibers showed a higher release in the first 10 min (74% and 72%). The high controlled release of sumac at 10 min and 60 min compared with that of curcumin can be related to the larger diameter of DKGS nanofibers. The high dissolution of curcumin at 10 min can be explained by the transformation of curcumin in the electrospinning process from a crystalline to an amorphous state, leading to an increase in its free energy [52]. In other studies, due to the low solubility of curcumin in aqueous media, in vitro curcumin release investigations were conducted in PBS (pH 7.4) containing Tween 80 (0.4–0.5% (*wt*/*v*)) and ethanol (10% *v*/*v*) [19,59]. The release of antimicrobial and antioxidant agents was also reported in 90:10 water:ethanol at different temperatures for polyelectrolyte multilayer (PEM) thin films loaded with 5% (*wt*/*v*) curcumin for transdermal drug delivery applications [60] and 70% ethanol in the case of clotrimazole-loaded fabric testing [61]. The sumac release from nanosheets was assessed at neutral (7.4) and acidic (4.5) pH values, simulating different microenvironment conditions in intact and injured skin [62].

The differences in the release of bioactive agents over time can be associated with the composition and surface-specific area of nanofibers, as well as the concentration of bioactive compounds.

### 2.5. In Vitro Cytotoxicity Evaluation

**Figure 6:**
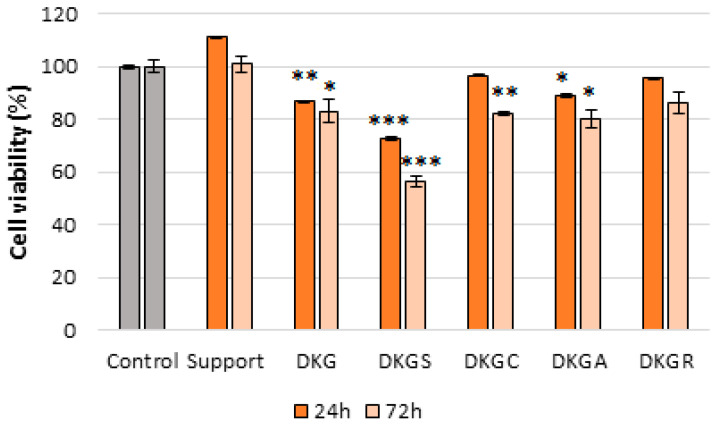
Viability of NCTC, clone L929, fibroblasts treated with Support, DKG, DKGS, DKGC, DKGA, and DKGR samples for 24 and 72 h, assessed by the MTT test. The control (untreated cells), to which all samples were reported, had 100% cell viability. Results were presented as mean values ± SD (*n* = 3). * means *p* ≤ 0.05, ** means *p* ≤ 0.01, and *** means *p* ≤ 0.001.

All samples based on keratin and gelatin from donkey hide did not show a cytotoxic effect (values of cell viability > 80%), except the DKGS sample (Figure 6). After 24 h of cell treatment, cell viability ranged between 111.04% (Support sample) and 86.74% (DKG sample), while the DKGS sample showed a slightly cytotoxic effect (cell viability of 72.58%). The same pattern was observed after 72 h, with all samples showing a good degree of cytocompatibility, with cell viability ranging between 100.88% (Support sample) and 80.16% (DKGA), except for the DKGS sample, where cell viability dropped significantly to 56.25% (Figure 6).

**Figure 7:**
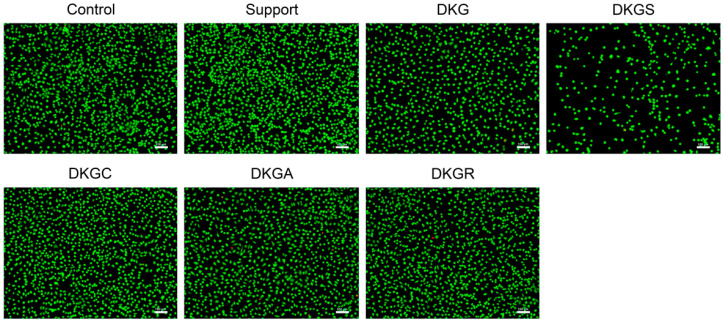
Fluorescence images of NCTC, clone L929, fibroblasts untreated (Control) and treated with Support, DKG, DKGS, DKGC, DKGA, and DKGR samples for 72 h; the Live/Dead test live cells labeled in green and dead cells labeled in red; scale bar = 100 µm.

The viability and morphology of NCTC, clone L929, cells treated with the different nanofibers were also evaluated by fluorescence microscopy after concomitant live and dead cell staining with two different dyes, namely calcein (green) and of dead cells with ethidium homodimer (red), respectively (Figure 7).

After 72 h of treatment with Support, DKGC, DKGA, and DKGR samples, the NCTC, clone L929, cells maintained their viability, morphological appearance, and cell density, being similar to the control sample (Figure 7).

**Figure 8:**
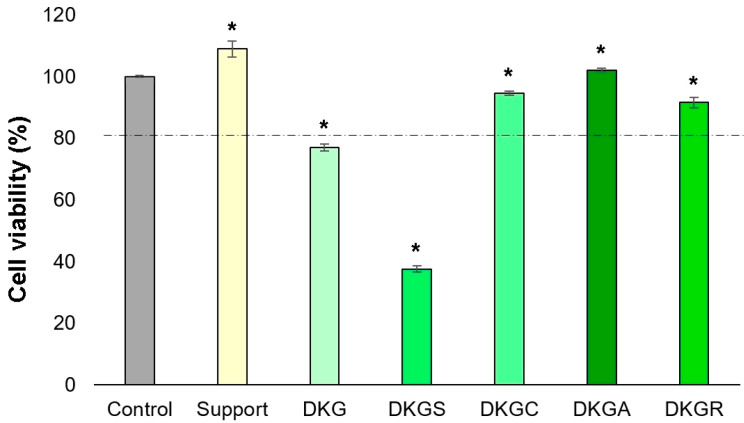
Viability of NCTC, clone L929, fibroblasts untreated (Control) and treated with Support, DKG, DKGS, DKGC, DKGA, and DKGR samples for 72 h, determined by the Live/Dead test. Results were reported as average value ± SD (*n* = 3). * *p* < 0.05 compared with the control sample.

Additionally, the insignificant proportion of dead cells indicated the cytocompatibility of the nanofibers. Statistical analysis indicated a 9% and 2% increase in cell viability for the Support and DKGA samples, compared to the control, while the DKGC and DKGR samples determined a 5% and 8% decrease in cell viability compared to the control (Figure 8). Outstanding cell viability and cell attachment capacity were also reported for some nanofibers containing acorn extract for mouse fibroblast (L929) cells [24]. Similar outcomes were found in the case of gelatin nanofibers enriched with propolis [50].

On the other hand, the DKG sample showed a slightly cytotoxic effect, and the DKGS sample had a moderate cytotoxic effect. Although the cells maintained their viability, the cell density decreased compared to the control sample. The cytotoxicity of DKGS nanofibers could be explained by the quantity of sumac extract (2% (*wt*/*v*)) added to the formulations. Previous studies had reported the cytotoxic effect of sumac extract at lower concentrations than the one used in the present study. Thus, the IC_50_ values of sumac methanolic extract tested on human umbilical vein endothelial cells (HUVEC) and retinoblastoma Y79 cells were 43 μg mL^−1^ and 9.1 μg mL^−1^, respectively [22,63]. In another study, the sumac extract showed no cytotoxic effect on HeLa cells at concentrations ranging between 31.25 μg mL^−1^ and 125 μg mL^−1^ after 48 h of treatment, whereas higher concentrations (250–2000 μg mL^−1^) induced a decrease in cell viability by around 30–65% compared to non-treated cells [64]. Batiha et al. [65] tested acetone extract of sumac on three various cell lines, namely mouse embryonic fibroblast (NIH/3T3), Madin-Darby bovine kidney (MDBK), and human foreskin fibroblasts (HFF), and the results showed that the extract inhibited the MDBK cells with half-maximal effective concentrations (EC_50_) of 737.7 μg mL^−1^, but did not reduce the HFF and NIH/3T3 cell viability at 1500 μg mL^−1^ [65]. Other authors also reported toxic effects for curcumin at concentrations above 150 μg mL^−1^ in methacrylated gelatin (GelMA) and methacrylated pectin (PeMA) hydrogels [66].

### 2.6. In Vitro Skin Wound Healing

**Figure 9:**
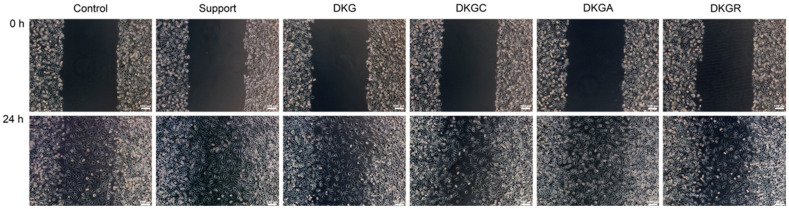
Optical microscopy photographs of the NCTC, clone L929, fibroblast monolayer after in vitro induction of a skin injury and application with the extraction medium of the Support, DKG, DKGC, DKGA, and DKGR samples for 24 h. Cell migration in the injured area can be observed. Scale bar = 100 µm.

**Figure 10:**
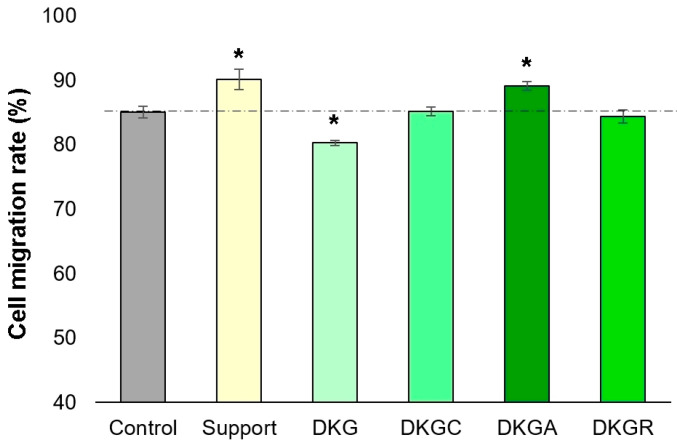
Migration rate (%) of NCTC, clone L929, fibroblasts treated with Support, DKG, DKGC, DKGA, and DKGR samples, for 24 h, evaluated with ImageJ software. Results were reported as the average value ± SD (*n* = 3). * *p* < 0.05 compared with the control sample.

An in vitro model of skin injury (scratch assay) was implemented to assess the ability of samples based on keratin and gelatin from donkey hide to accelerate the proliferation and migration of cells and to cover the injured area, and therefore to induce the healing of a “wound”. Thus, the DKGA sample presented the highest migration rate (89%) after 24 h, with 4% more than the control (85%), followed by the DKGC (85%) and DKGR (84%) samples, which had a migration rate similar to that of the control sample, and by the DKG sample with a migration rate of 80% (Figure 9 and Figure 10). In conclusion, the DKGA sample was the most efficient in repairing the injured cell monolayer after 24 h of treatment, promoting cell proliferation and migration.

### 2.7. Assessment of the Antimicrobial Activity of Donkey Gelatin-Based Nanofibers Loaded with Plant Extracts

Table 5 and Table 6 show that the plant-based extracts reduced the bacterial load of donkey gelatin and keratin nanofibers to an acceptable level for topical and pharmaceutical products.

TAMC (total aerobic microbe count) and TYMC (total yeast and mold count) are the strains naturally developed on the nanofiber surfaces under favorable conditions (nutrient medium, temperature) for bacteria or fungi, respectively.

Nanofibers with curcumin extract showed very similar antimicrobial and antifungal performances to nanofibers with rivanol (the reference sample), while nanofibers with acorn and sumac extracts demonstrated similar values for TAMC (10–11 CFU/g) and TYMC (3.33–4.33 CFU/g). According to the Pharmacopoeia criteria, the results allow the use of donkey gelatin-based nanofibers with plant extracts and rivanol as topical or pharmaceutical products [67]. It is obvious that the plant extracts and rivanol improved the antimicrobial and antifungal properties of donkey gelatin and keratin nanofibers.

In a similar paper, the nanofibers loaded with acorn extract exhibited a 90% antibacterial activity against the *Staphylococcus aureus* bacterium, as determined by the quantitative standard test method [23].

## 3. Conclusions

To the authors’ knowledge, the gelatin and keratin extracted from donkey hide were exploited for the first time to fabricate nanofibrous wound dressings. The bioactive extracts of sumac, acorn, and curcumin added to gelatin/keratin nanofibers contributed to the enhancement of antioxidant activity and the obtaining of nanofibers that mimic the conditions of ideal wound dressings. Biocompatibility and healing properties depend on the concentration of bioactive extracts. Further studies are needed to find the correlation between the concentration of natural extracts and the in vitro biocompatibility of donkey keratin gelatin nanofibers loaded with antioxidant agents.

## 4. Materials and Methods

### 4.1. Materials

Donkey hide and hair were acquired from a Romanian animals’ farm for scientific purposes. Oak acorn was purchased from the Serbian market, sumac from Turkish suppliers, curcumin (1,7-bis(4-hydroxy-3-methoxyphenyl)-1,6-heptadiene-3,5-dione), and rivanol (2-ethoxy-6,9-diaminoacridine monolactate) 0.1% (*wt*/*v*) were purchased from the Romanian market. Poly(ethylene) oxide (PEO) was used in the form of powder (MW of 100,000, Alfa Aesar, Kandel, Germany).

Reagents used in the microbiological study were: tryptone soy agar (TSA), tryptic soy broth (TSB), enumeration agar (EA), soybean casein digest lecithin polysorbate 80 (SCDLP), nutrient broth (NB), and Sabouraud dextrose agar (SDA). *Escherichia coli* ATCC 10536, *Staphylococcus aureus* ATCC 6538, and *Candida albicans* ATCC 10231 were used as microorganism strains. All microbiology reagents and microorganism strains were acquired from Mediclim, Otopeni, Romania. All chemicals employed were of reagent grade.

### 4.2. Preparation of Gelatin and Keratin Loaded with Bioactive Agents

**Figure 11:**
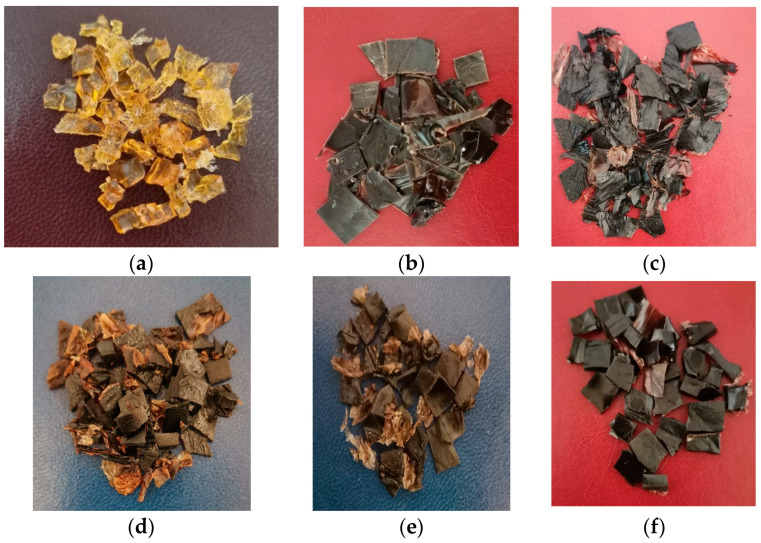
Gelatin, gelatin/keratin, and bioactive gelatin/keratin extracted from donkey hide. (**a**) Gelatin; (**b**) DKG; (**c**) DKGA; (**d**) DKGC; (**e**) DKGS; (**f**) DKGR.

Donkey hide was processed to remove impurities, interfibrillar substances, and hair, until the delimed hide stage; then 100 g pelt was successively washed to remove soluble salts, shredded in a mincer, immersed in water at a ratio of 350 wt%, and heated in a water bath at a temperature of 90 °C for 5–16 h. The resulting extract was separated by residue using a stainless-steel sieve with pores size < 150 μm, followed by cooling and drying in an oven at 60 °C, when gelatin granules were obtained (Figure 11a). Keratin was obtained from 100 g of donkey hair by heating to 80 °C in a solution of 1.5% (*wt*/*v*) NaOH for 5 h, then it was filtered and dried. Donkey gelatin-keratin (DKG) was obtained by mixing the original solutions (Table 1) in equal proportions (1:1% *v*/*v*) and drying in an oven at 60 °C, resulting in a solid composite with an estimated composition of 72% gelatin and 28% keratin (Figure 11b).

The plant extracts were prepared by heating the plants at 90 °C for 4 h at water in a concentration of 4% (*wt*/*v*), higher than their minimum inhibitory concentration values (Table 7).

Equal volumes of each plant extract type were added to the gelatin/keratin solution (DKGS, DKGA, and DKGA), followed by drying at 60 °C (Figure 11c,d). A 10% (*v*/*v*) solution of rivanol was introduced to the gelatin/keratin (DKGR) dispersion and used as a control (Figure 11f).

### 4.3. Characterization of Gelatin and Gelatin/Keratin Loaded with Bioactive Agents

The extracted gelatin and gelatin/keratin were investigated using physical-chemical methods, as follows: determination of dry matter, conducted in accordance with EN ISO 4684 [68], evaluation of pH levels following the guidelines outlined in STAS 8619/3 [69], and examination of electrical conductivity based on EN ISO 27883 [70]. Total dissolved solids and salinity (the content of salts) were estimated as indirect measurements from electrical conductivity. These physical-chemical parameters were evaluated using a conductivity (C1010, Consort Turnhout, Belgium) and pH meter (Consort C831 Multiparameter analyzer, Turnhout, Belgium). The gelatin strength and relaxation of donkey and donkey gelatin mixed with donkey keratin (DKG) were determined by using the TEX’AN TOUCH 50 N texture analyzer (LAMY RHEOLOGY, Champagne au Mont d’Or, France) for 6.67% solution after cooling for 16–18 h at 10.0 ± 0.1 °C according to Gelatin Manufacturers Institute of America standard [71]. Rheological parameters such as viscosity, shear stress, and shear rate were conducted using a Brookfield AMETEK DV2T Viscometer (Middleboro, MA, USA) with a spindle No. 21. Furthermore, the diameter and polydispersity index of gelatin/keratin loaded with bioactive agents were assessed using the Dynamic Light Scattering (DLS) method with the help of a Zetasizer Nano-ZSP device, which operated at λ = 633 nm, and a light source of He-Ne laser (Malvern Instruments Limited, Worcestershire, UK). An amount of 0.1 g of each granule was immersed in 5 mL of ultrapure water and subjected to sonication for 5 min. Subsequently, three drops of the resulting suspension were introduced into a 10 mL solution of 1 mM NaCl. This mixture was then thoroughly homogenized and analyzed using a 12 mm cell (DTS 0012). Zeta potential was determined using the electrophoretic technique (cell DTS 1070).

### 4.4. Preparation of Bioactive Nanofibers and Analysis of Their Structure

20 g of gelatin-keratin granules extracted from donkey hide and hair loaded with an antioxidant extract such as sumac, curcumin, or acorn shell, a having ratio between gelatin:keratin:bioactive extract of 71:27:2 wt%, were dissolved in 100 mL of distilled water by stirring on a magnetic plate at a temperature of 50 °C and 400 rpm. Then, the homogeneous solutions were centrifuged for 3 min at 60 × 100 rpm. 10 mL of supernatant was mixed with 10 mL of a 10% (*wt*/*v*) solution of PEO. This solution was filled into a 20 mL Teflon syringe fitted with a tube and a G21-gauge metal needle attached to the other end within the electrospinning equipment (TL Pro-BM, Tong Li Tech Co., Ltd., Bao An, Shenzhen, China). The electrospinning technique took place at an ambient temperature of 22.6 °C and a relative humidity of 40%. The resulting nanofibers were collected on a drum covered with polypropylene mesh for medical use, denoted as support. Gelatin/keratin with rivanol was processed into nanofibers in a similar way to bioactive agents and used as control nanofibers with recognized antimicrobial activity. The electrospinning parameters are presented in Table 8.

This process of obtaining bioactive nanofibers is simple, versatile, reproducible, and occurs at room temperature without high energy consumption or the use of potentially toxic solvents. Additionally, environmental sustainability is ensured by valuing existing protein resources and natural biocompounds.

The morphology, fiber size diameters, and elemental compositions of fabricated bioactive nanofibers were investigated by using scanning electron microscopy (SEM)/energy dispersive X-ray spectrometry (EDS) analysis (FEI, QUANTA 450 FEG, Eindhoven, The Netherlands). SEM images were captured using an FEI Inspect S50 Scanning Electron Microscope. To mitigate the effect of charging, a thin gold layer was applied to all samples using a Cressington 108 auto sputter coater equipped with a Cressington mtm 20 thickness controller. Secondary electron imaging was obtained at a length of 10 mm, employing an acceleration voltage of 10 kV and magnifications of 50× and 10,000×, respectively. The mean thickness was determined by measuring the diameter of at least 50 nanofibers without beads and calculating the average using Origin Pro 21 and ImageJ software version 1.54d.

### 4.5. Antioxidant Activity of Bioactive Nanofibers

Antioxidant activity was determined both for plants and nanofibers containing protein and bioactive extracts by keeping in contact with the 2,2-azino-bis-3-ethylbenzthiazoline-6-sulphonic acid radical cation (ABTS^•+^) at 738 nm, according to the method described in [72]. Sumac and curcumin plants, in powder form, were dissolved in ethanol, while that of keratin was dissolved in distilled water. Their concentrations were 4000 µg mL^−1^. The standards were prepared at various concentrations between 3.2–16 µg mL^−1^ for sumac extract, 12.8–160 µg mL^−1^ in the case of curcumin extract, and 16–160 µg mL^−1^ in the case of keratin extract. For the radical scavenging activity (RSA, %) assay, 20 µL from each known concentration of natural plants were mixed with a 4 mL solution of ABTS^•+^ and incubated in the dark for 6 min. After that, their absorbance was measured at 738 nm in comparison to a blank using an ultraviolet-visible spectrometer (Orion UV-Vis AQUAMATE 8000, Thermo Fisher Scientific, Waltham, MA, USA). A 2 × 1.5 cm^2^ surface of bioactive nanofibers deposited on a PP support, containing an amount of nanofibers in the range of 6.9 mg to 11.1 mg, was combined with a 4 mL solution of ABTS^•+^, and the absorbance was measured spectrophotometrically after 10 min.

Radical scavenging activity (*RSA*, %) was calculated according to Equation (1).

(1) $$\%RSA=\left(\frac{{Abs}_{blank}-{Abs}_{sample}}{{Abs}_{blank}}\right)\times 100$$

IC_50_ values represent the maximum active compounds from bioactive agents needed to deactivate 50% of a given amount of ABTS^•+^. Thus, low IC_50_ values indicate a higher level of antiradical efficiency.

The analyses were performed in triplicate, and the results are reported as the mean value ± standard deviation.

### 4.6. Controlled Release of Sumac and Curcumin

About 0.2 g of nanofibers containing sumac and curcumin (DKGS, DKGC) were immersed in 10 mL of 70 wt% ethanol. At intervals of 10, 30, and 60 min after sonication, an aliquot (4 mL) was taken and exposed to centrifugation with a speed of 4000 rpm and a time of 3 min. The absorbance of the supernatant was read at 270 nm for DKGS and 425 nm for DKGC, characteristic of the π-π * electronic transition, using a UV-Vis spectrophotometer. The controlled releases for sumac and curcumin were calculated according to the calibration curves obtained for 10, 50, 10, 200, and 300 ppm of sumac solution and 50, 100, and 500 ppm of curcumin solution in ethanol.

The percentage of sumac and curcumin extracts released from the nanofibers was determined using Equation (2).

(2) $$\%\;Release=\frac{The\;amount\;of\;released\;extracts\;at\;a\;specific\;time}{Calculated\;amount\;of\;extract\;in\;nanofibers}\times 100$$

### 4.7. In Vitro Cytotoxicity Assessment

NCTC, clone L929, and murine fibroblasts were used to assess the in vitro cytotoxicity of the nanofibers according to the [73]. All samples were cut into discs of (5 × 5) mm^2^ and sterilized with UV light for 4 h.

The cell viability and morphology were assessed by the quantitative MTT assay and by fluorescence microscopy using the Live/Dead assay. The NCTC fibroblasts were seeded in MEM culture medium at a cell density of 5 × 10^4^ cells/mL in 24-well culture plates and incubated overnight at 37 °C in a humid atmosphere with 5% CO_2_ to allow cell adhesion. After 24 h, the nanofibers were added (1 disc/well) in fresh culture medium, and cells were further incubated for 24 and 72 h, respectively. After this period, the cells were incubated for 3 h at 37 °C with MTT solution (0.25 mg mL^−1^), after which the insoluble formazan crystals were dissolved in isopropanol. The plates were for incubated 15 min at room temperature with gentle shaking for color uniformity, after which the absorbance was measured at 570 nm using a Tecan Sunrise plate reader (Tecan, Austria). The obtained values are directly proportional to the number of living cells present at the end of the incubation. The results were reported as percentages of viability compared to the control sample (non-treated cells), considered to have 100% viability. All samples were evaluated in triplicate.

A Live/Dead assay kit (Molecular Probes, Invitrogen, Eugene, OR, USA) was used to evaluate cell morphology and viability according to the manufacturer’s instructions. The assay is based on the concomitant staining of live cells (green) and dead cells (red) with two specific reagents, namely calcein AM and ethidium homodimer-1, respectively. After treatment with the nanofibers for 72 h, the cells were stained with 2 μM calcein-AM (2 μM) and 4 μM ethidium homodimer-1 for 30 min at room temperature. A Zeiss Axio Observer D1 microscope was used to acquire the fluorescent images, which were further processed with ImageJ 1.51 software.

### 4.8. In Vitro Wound Healing Assay (Scratch Assay)

This method was used to investigate the capacity of tested samples to induce cell proliferation and migration into an injured cell monolayer. For this assay, only the samples that presented values of cell viability higher than 80% based on the quantitative MTT test were selected. Thus, NCTC, clone L929, and murine fibroblasts were cultivated at a cell density of 3 × 10^5^ cells/mL and maintained at 37 °C in a humid atmosphere with 5% CO_2_, until a cellular monolayer was obtained. Subsequently, a linear wound was created with a sterile pipette tip in the cell monolayer, the sample extracts (sample incubation in MEM medium for 24 h at 37 °C) were added, and cells were incubated for another 24 h. Photographs were taken with an Axio Observer D1 microscope (Carl Zeiss, Oberkochen, Germany) at the beginning of the experiment (T0) and after 24 h of cell incubation in order to assess the cell migration rate. The ImageJ 1.51 software was used to quantify the percentage of cell migration into the injured area. Samples were run in triplicate. The data were presented as the mean value ± standard deviation of the recovery rates of the injured area. Statistical analyses were performed using the Student’s t-test, with differences considered statistically significant at *p* ≤ 0.05.

### 4.9. Evaluation of Microbial Contamination

The control of microbial contamination aims to assess the total number of aerobic microorganisms or the absence of pathogenic or conditioned-pathogenic microorganisms. Bacteria or fungi were sampled from the preserved stock to reach the initial concentration. A plate with EA was streaked and incubated at 37 °C ± 20 °C for 24 h to 48 h, then 20 mL of TSB was added in a 100 mL Erlenmeyer flask. The initial cell concentration had been established through decimal dilutions (10^5^), and in the final dilution, 100 µL was extracted and spread onto nutrient agar for each strain. Plate counts were conducted after 24 h of incubation and used as a reference for cell growth in both control and test samples. Therefore, plates with cell densities similar to those of the 10^5^ dilutions were considered to have comparable CFU values (1.2 × 10^5^ CFU/g for *Staphylococcus aureus*, 1 × 10^5^ CFU/g for *Escherichia coli*, and 2.5 × 10^4^ CFU/g for *Candida albicans*). Subsequently, 1.0 ± 0.1 mL of the inoculum was pipetted at several points over each test sample, which was then placed in vials. After inoculation, the vials were shaken, and 20 mL of SCDLP medium was immediately added. The vials containing the test material were incubated at 37 ± 1 °C for 18–24 h. Then, 1 mL of the inoculum from the bacterial suspension was taken from the sample, placed in a test tube containing 9.0 mL ± 0.1 mL of NB, and shaken well. 1 mL of this solution was added to another test tube containing 9.0 mL ± 0.1 mL of medium and shaken well. These operations were performed successively 10 times to prepare a series of dilutions. Subsequently, 1 mL of each dilution was pipetted into two Petri dishes, and 15 mL of TSA, heated to 45 °C ± 1 °C in a water bath, was added to the Petri dishes for the enumeration of colony-forming units (CFU). Plates with no more than 250 colonies for bacteria and 50 colonies for yeasts and filamentous fungi were chosen for a correct evaluation. The developed colonies were taken into consideration, and the average was made for each dilution and expressed as the total number of CFU per g. Results were expressed as total bacterial count (TAMC), which is the mean CFU determined on casein and soy hydrolysate agar medium, and total yeast and filamentous fungi (TYMC), which is the mean CFU evaluated on Sabouraud agar medium with chloramphenicol.

The sterility test was performed by the filtration method using a concentration of 10^6^ CFU/mL of *Staphylococcus aureus*, *Escherichia coli,* or *Candida albicans*, according to [74]. 

### 4.10. Statistical Analysis

For the radical scavenging activity (RSA) assay of the bioactive DKG nanofibers, cell viability, and migration rate tests, a one-way Anova test was used to find a statistically significant difference between groups at a significance level (*p* ≤ 0.05). The results reporting physical-chemical characteristics of bioactive formulations, average diameter of nanofiber measured by SEM, controlled release of nanofibers, and minimum inhibitory concentration of bioactive extracts were expressed as means ± standard deviation.

## Figures and Tables

**Table 1:** Characteristics of gelatin and gelatin/keratin extracted from donkey hide and donkey hair.

Characteristics	Gelatin	Keratin	Gelatin/Keratin
Dry matter content (%)	11.89 ± 0.35	14.30 ± 0.35	13.00 ± 0.35
pH (1:10) (pH units)	7.20 ± 0.10	10.30 ± 0.10	9.2 ± 0.10
Gel strength (g)	321.00 ± 2.45	-	421.00 ± 3.50
Relaxation (%)	21.50 ± 0.50	-	17.40 ± 0.45

**Table 2:** Characteristics of gelatin/keratin extracted from donkey hide loaded with bioactive extracts.

Property	DKG *	DKGS **	DKGC **	DKGA **	DKGR ***
pH	9.20 ± 0.50	7.10 ± 0.30	9.10 ± 0.50	7.50 ± 0.10	9.00 ± 0.50
Electrical conductivity (mS/cm)	0.508 ± 0.04	9.45 ± 0.85	12.95 ± 0.95	10.84 ± 0.90	10.14 ± 0.85
Total dissolved solids (mg/L)	225 ± 15	5200 ± 24	693 ± 20	5910 ± 15	5560 ± 14
Salinity (ppt)	0	5.3 ± 0.1	7.4 ± 0.1	6.1 ± 0.1	5.7 ± 0.1
Viscosity (cP) at 28.1 °C	22	647	2330	2500	90
Speed (RPM)	200	50	10	200	200
Shear Stress (dyne/cm^2^)	40.92	300.90	216.70	230.60	167.40
Shear Rate (1/s)	186.0	46.5	9.3	186.0	186.0

* 28.00 wt% keratin, 72.00 wt% gelatin; ** 27.00 wt% keratin, 71.00 wt% gelatin, and 2.00 wt% plant extract; *** 28.00 wt% keratin, 71.90 wt% gelatin, and 0.1 v% rivanol.

**Table 3:** Elemental compositions of the fabricated bioactive nanofibers.

Element	DKG		DKGS		DKGC		DKGA		DKGR	
	Weight (%)	Atomic (%)	Weight (%)	Atomic (%)	Weight (%)	Atomic (%)	Weight (%)	Atomic (%)	Weight (%)	Atomic (%)
Carbon (C)	43.6	65.33	37.53	61.74	32.33	50.67	19.02	49.23	44.28	65.2
Nitrogen (N)	16.78	21.56	15.3	21.59	15.09	20.28	6.11	13.55	16.69	21.08
Oxygen (O)	8.08	9.09	8.02	9.9	11.47	13.5	3.72	7.23	8.26	9.13
Natrium (Na)	1.62	1.27	2.4	2.06	8.44	6.91	2.7	3.65	1.82	1.4
Calcium (Ca)	-	-	2.61	1.29	9.6	5.1	-	-	-	-
Sulfur (S)	-	-	-	-	0.96	0.56	-	-	0.67	0.37
Chlorine (Cl)	-	-	-	-	9.6	5.1	3.1	2.76	-	-

**Table 4:** Radical scavenging activity (*RSA*) assay for the bioactive DKG nanofibers.

Samples	*RSA* (%)
DKG	87.69 ± 1.11 (*p* < 0.01)
DKGS	89.09 ± 0.37 (*p* < 0.001)
DKGC	89.00 ± 7.89 (*p* < 0.01)
DKGA	80.01 ± 19.37 (*p* > 0.05)
DKGR	87.43 ± 6.41 (*p* < 0.01)

**Table 5:** Total aerobic microbial count (TAMC) and total yeast and mold count (TYMC) of nanofibers.

Nanofiber Samples	TAMC (CFU/g)	TYMC (CFU/g)
DKG	1.76 × 10^2^	57.00
DKGS	11.00	3.33
DKGC	1.00	0
DKGA	10.00	4.33
DKGR	0	0

**Table 6:** The sterility test against *S. aureus*, *E. coli,* and *C. albicans*.

Nanofiber Samples	*S. aureus*	*E. coli*	*C. albicans*
DKG	Absent	Absent	Absent
DKGS	Absent	Absent	Absent
DKGC	Absent	Absent	Absent
DKGA	Absent	Absent	Absent
DKGR	Absent	Absent	Absent

**Table 7:** Minimum inhibitory concentrations of curcuma, oak acorn, sumac, and rivanol (5 ug mL^−1^).

Bacteria	Curcuma	Oak acorn	Sumac	Rivanol
	mL			
*S. aureus*	9.58 ± 0.07	6.33 ± 0.98	7.90 ± 0.40	0.63 ± 0.30
*E. coli*	12.68 ± 0.08	4.50 ± 0.22	6.40 ± 0.20	0.16 ± 0.22

**Table 8:** Electrospinning parameters for fabrication of bioactive donkey keratin/gelatin nanofibers containing 0.75% gelatin, 0.875% keratin, and 0.25% bioactive agent related to the electrospinning solution.

Nanofiber Sample	Code	Electrospinning Parameters		
		Flow (mL/h)	Voltage (kV)	Distance between Needle to Collector (cm)
Keratin/Gelatin extract	DKG	1.1	22.07	13
Keratin/Gelatin/Sumac extract	DKGS	0.5	22.07	13
Keratin/Gelatin/Curcumin extract	DKGC	1.0	22.58	13
Keratin/Gelatin/Acorn extract	DKGA	1.0	21.84	13
Keratin/Gelatin/Rivanol	DKGR	0.7	23.24	13

## Data Availability

The data presented in this study are openly available in the article.

## References

[1] Guo S., DiPietro L.A. (2010). Factors Affecting Wound Healing. J. Dent. Res..

[2] Haroun M. (2022). Review on the Developments of Benzothiazole-containing Antimicrobial Agents. Curr. Top. Med. Chem..

[3] Muteeb G., Rehman M.T., Shahwan M., Aatif M. (2023). Origin of Antibiotics and Antibiotic Resistance, and Their Impacts on Drug Development: A Narrative Review. Pharmaceuticals.

[4] McCann M.T., Gilmore B.F., Gorman S.P. (2008). *Staphylococcus epidermidis* device-related infections: Pathogenesis and clinical management. J. Pharm. Pharmacol..

[5] Guo N., Xia Y., Zeng W.S., Chen J., Wu Q.X., Shi Y.X., Li G.Y., Huang Z.Y., Wang G.H., Liu Y. (2022). Alginate-based aerogels as wound dressings for efficient bacterial capture and enhanced antibacterial photodynamic therapy. Drug Deliv..

[6] Darie-Niță R.N., Râpă M., Frąckowiak S. (2022). Special Features of Polyester-Based Materials for Medical Applications. Polymers.

[7] Fleck C.A., Simman R. (2010). Modern collagen wound dressings: Function and purpose. J. Am. Coll. Certif. Wound Spec..

[8] Ehrlich F., Lachner J., Hermann M., Tschachler E., Eckhart L. (2020). Convergent Evolution of Cysteine-Rich Keratins in Hard Skin Appendages of Terrestrial Vertebrates. Mol. Biol. Evol..

[9] Konop M., Rybka M., Drapala A. (2021). Keratin Biomaterials in Skin Wound Healing, an Old Player in Modern Medicine: A Mini Review. Pharmaceutics.

[10] Wang D., Ru W., Xu Y., Zhang J., He X., Fan G., Mao B., Zhou X., Qin Y. (2014). Chemical constituents and bioactivities of *Colla corii asini*. Drug Discov. Ther..

[11] Liang R., Xu L., Fan C., Cao L., Guo X. (2023). Structural Characteristics and Antioxidant Mechanism of Donkey-Hide Gelatin Peptides by Molecular Dynamics Simulation. Molecules.

[12] Kim J.-S., Kim D., Kim H.-J., Jang A. (2018). Protection effect of donkey hide gelatin hydrolysates on UVB-induced photoaging of human skin fibroblasts. Process Biochem..

[13] Bernardo M.P., Pasquini D., Mattoso L.H.C. (2023). Enhanced antibacterial activity of wound dressings based on alginate/hydroxyapatite modified with copper and naproxen. J. Mater. Res..

[14] Mouro C., Gouveia I.C. (2023). Electrospun wound dressings with antibacterial function: A critical review of plant extract and essential oil incorporation. Crit. Rev. Biotechnol..

[15] Sayyar Z., Hosseini Z., Beheshtizadeh N. (2024). Developing curcumin loaded-magnetic montmorillonite nanoparticles/polyvinyl alcohol/hyaluronic acid/chitosan nanofiber mats as a wound dressing. J. Drug Deliv. Sci. Technol..

[16] Falbo F., Spizzirri U.G., Restuccia D., Aiello F. (2023). Natural Compounds and Biopolymers-Based Hydrogels Join Forces to Promote Wound Healing. Pharmaceutics.

[17] Bai Q., Hu F.F., Gou S.Y., Gao Q., Wang S.H., Zhang W.H., Zhang Y.N., Lu T.L. (2024). Curcumin-loaded chitosan-based hydrogels accelerating *S. aureus*-infected wound healing. Int. J. Biol. Macromol..

[18] Singh H., Dhanka M., Yadav I., Gautam S., Bashir S.M., Mishra N.C., Arora T., Hassan S. (2023). Technological Interventions Enhancing Curcumin Bioavailability in Wound-Healing Therapeutics. Tissue Eng. Part B Rev..

[19] Uyar M., Cakmak S. (2024). Three-dimensional macro/micro-porous curcumin releasing polycaprolactone/chitosan nanofiber scaffolds as wound dressing. Colloids Surf. A Physicochem. Eng. Asp..

[20] Cheng C., Wang R., Ma J., Zhang Y., Jing Q., Lu W. (2024). Examining the wound healing potential of curcumin-infused electrospun nanofibers from polyglutamic acid and gum arabic. Int. J. Biol. Macromol..

[21] Dalar A., Dogan A., Bengu A.S., Mukemre M., Celik I. (2018). Screening in vivo antioxidant and haematological properties of sumac and acorn bioactive rich extracts. Ind. Crops Prod..

[22] Alsamri H., Athamneh K., Pintus G., Eid A.H., Iratni R. (2021). Pharmacological and Antioxidant Activities of *Rhus coriaria* L. (Sumac). Antioxidants.

[23] Hadipour-Goudarzi E., Hemmatinejad N., Shokrgozar M.A. (2023). Fabrication of Acorn-Loaded Chitosan/Gelatin Nanofibrous Web to Increase Antibacterial Activity for Wound-Healing Applications. Fibers Polym..

[24] Gabr S.A., Alghadir A.H. (2019). Evaluation of the Biological Effects of Lyophilized Hydrophilic Extract of *Rhus coriaria* on Myeloperoxidase (MPO) Activity, Wound Healing, and Microbial Infections of Skin Wound Tissues. Evid.-Based Complement. Altern. Med..

[25] Martins R.B., Gouvinhas I., Nunes M.C., Peres J.A., Raymundo A., Barros A. (2020). Acorn Flour as a Source of Bioactive Compounds in Gluten-Free Bread. Molecules.

[26] Burlacu E., Nisca A., Tanase C. (2020). A Comprehensive Review of Phytochemistry and Biological Activities of *Quercus* Species. Forests.

[27] Zhang X.T., Wang Y.X., Gao Z.Y., Mao X.H., Cheng J.X., Huang L.J., Tang J.G. (2023). Advances in wound dressing based on electrospinning nanofibers. J. Appl. Polym. Sci..

[28] Katrilaka C., Karipidou N., Petrou N., Manglaris C., Katrilakas G., Tzavellas A.N., Pitou M., Tsiridis E.E., Choli-Papadopoulou T., Aggeli A. (2023). Freeze-Drying Process for the Fabrication of Collagen-Based Sponges as Medical Devices in Biomedical Engineering. Materials.

[29] Gaidau C., Rapa M., Stefan L.M., Matei E., Berbecaru A.C., Predescu C., Mititelu-Tartau L. (2023). Conversion of Animal-Derived Protein By-Products into a New Dual-Layer Nanofiber Biomaterial by Electrospinning Process. Fibers.

[30] Berechet M.D., Gaidau C., Nesic A., Constantinescu R.R., Simion D., Niculescu O., Stelescu M.D., Sandulache I., Rapa M. (2023). Antioxidant and Antimicrobial Properties of Hydrolysed Collagen Nanofibers Loaded with Ginger Essential Oil. Materials.

[31] Matei E., Gaidau C., Rapa M., Stefan L.M., Ditu L.M., Predescu A.M., Stanca M., Pantilimon M.C., Berechet M.D., Predescu C. (2021). Sustainable Coated Nanostructures Based on Alginate and Electrospun Collagen Loaded with Antimicrobial Agents. Coatings.

[32] Rapa M., Gaidau C., Mititelu-Tartau L., Berechet M.D., Berbecaru A.C., Rosca I., Chiriac A.P., Matei E., Predescu A.M., Predescu C. (2021). Bioactive Collagen Hydrolysate-Chitosan/Essential Oil Electrospun Nanofibers Designed for Medical Wound Dressings. Pharmaceutics.

[33] Gaidau C., Rapa M., Stanca M., Tanase M.-L., Olariu L., Constantinescu R.R., Lazea-Stoyanova A., Alexe C.-A., Tudorache M. (2023). Fish Scale Gelatin Nanofibers with *Helichrysum italicum* and *Lavandula latifolia* Essential Oils for Bioactive Wound-Healing Dressings. Pharmaceutics.

[34] Gaidau C., Rapa M., Ionita G., Stanculescu I.R., Zaharescu T., Constantinescu R.-R., Lazea-Stoyanova A., Stanca M. (2024). The Influence of Gamma Radiation on Different Gelatin Nanofibers and Gelatins. Gels.

[35] Râpă M., Gaidău C., Stefan L.M., Matei E., Niculescu M., Berechet M.D., Stanca M., Tablet C., Tudorache M., Gavrilă R. (2020). New nanofibers based on protein by-products with bioactive potential for tissue engineering. Materials.

[36] Su K., Sun W., Li Z., Huang T., Lou Q., Zhan S. (2023). Complex Modification Orders Alleviate the Gelling Weakening Behavior of High Microbial Transglutaminase (MTGase)-Catalyzed Fish Gelatin: Gelling and Structural Analysis. Foods.

[37] Xue H., Xu M., Zhang G., Wang P., Yu L., Zhao Y., Tu Y., Zhao Y. (2022). Study on the mechanism of enhanced gel strength of heat-induced egg white by shikimic acid braising. Poult. Sci..

[38] Mikhailov O.V. (2023). Gelatin as It Is: History and Modernity. Int. J. Mol. Sci..

[39] Rather J.A., Akhter N., Ashraf Q.S., Mir S.A., Makroo H.A., Majid D., Barba F.J., Khaneghah A.M., Dar B.N. (2022). A comprehensive review on gelatin: Understanding impact of the sources, extraction methods, and modifications on potential packaging applications. Food Packag. Shelf Life.

[40] White Powder Unflavored Donkey Hide Gelatin Powder. https://nutrition-powders.com/sale-13832501-white-powder-unflavored-donkey-hide-gelatin-powder-einecs-no-232-554-6.html.

[41] Bulk Gelatin & Collagen. https://customcollagen.com.

[42] Dinçer M.T., Erdem Ö.A., Kalkan H., Üçok M.Ç. (2016). Comparison of recovered carp scales (*Cyprinus carpio*) gelatin and commercial calf and pork skin gelatins. Ege J. Fish. Aquat. Sci..

[43] Osorio F.A., Bilbao E., Bustos R., Alvarez F. (2007). Effects of concentration, bloom degree, and pH on gelatin melting and gelling temperatures using small amplitude oscillatory rheology. Int. J. Food Prop..

[44] Wang S.A., Zhu F. (2017). Chemical composition and biological activity of staghorn sumac (*Rhus typhina*). Food Chem..

[45] Matthaus B., Özcan M.M. (2015). Fatty acid composition, tocopherol, and sterol contents of sumac (*Rhus coriaria* L.) fruit oils. Eur. J. Lipid Sci. Technol..

[46] Papoti V.T., Kizaki N., Skaltsi A., Karayannakidis P.D., Papageorgiou M. (2018). The phytochemical rich potential of acorn (*Quercus aegilops*) products and by products. Food Sci. Biotechnol..

[47] Jabborova D., Choudhary R., Karunakaran R., Ercisli S., Ahlawat J., Sulaymanov K., Azimov A., Jabbarov Z. (2021). The Chemical Element Composition of Turmeric Grown in Soil-Climate Conditions of Tashkent Region, Uzbekistan. Plants.

[48] Shahrivari S., Zeebaree S.M.S., Alizadeh-Salteh S., Feizy H.S., Morshedloo M.R. (2024). Phytochemical variations antioxidant, and antibacterial activities among zebaria sumac (*Rhus coriaria* var. zebaria) populations in Iraq. Sci. Rep..

[49] Mouro C., Martins R., Gomes A.P., Gouveia I.C. (2023). Upcycling Wool Waste into Keratin Gel-Based Nanofibers Using Deep Eutectic Solvents. Gels.

[50] Al-Sudani B.T., Mahmoudi E., Shaker Al-Naymi H.A., Al-Musawi M.H., Al-Talabanee I.B.N., Ramezanis S., Ghorbani M., Moghadam F.M. (2024). Antibacterial and wound healing performance of a novel electrospun nanofibers based on polymethyl-methacrylate/gelatin impregnated with different content of propolis. J. Drug Deliv. Sci. Technol..

[51] Zhou X., Liu Y., Wang X., Li X., Xiao B. (2020). Effect of particle size on the cellular uptake and anti-inflammatory activity of oral nanotherapeutics. Colloids Surf. B Biointerfaces.

[52] Gezici S., Sekeroglu N. (2019). Neuroprotective potential and phytochemical composition of acorn fruits. Ind. Crops Prod..

[53] Pinto D., Franco S.D., Silva A.M., Cupara S., Koskovac M., Kojicic K., Soares S., Rodrigues F., Sut S., Dall’Acqua S. (2019). Chemical characterization and bioactive properties of a coffee-like beverage prepared from *Quercus cerris* kernels. Food Funct..

[54] Gaidau C., Epure D.-G., Enascuta C.E., Carsote C., Sendrea C., Proietti N., Chen W., Gu H. (2019). Wool keratin total solubilisation for recovery and reintegration—An ecological approach. J. Clean. Prod..

[55] Zannou O., Oussou K.F., Chabi I.B., Alamou F., Awad N.M.H., Miassi Y.E., Loke F.C.V., Abdoulaye A., Pashazadeh H., Redha A.A. (2025). Phytochemical and nutritional properties of sumac (*Rhus coriaria*): A potential ingredient for developing functional foods. J. Future Foods.

[56] Zheng Q.T., Yang Z.H., Yu L.Y., Ren Y.Y., Huang Q.X., Liu Q., Ma X.Y., Chen Z.K., Wang Z.B., Zheng X. (2017). Synthesis and antioxidant activity of curcumin analogs. J. Asian Nat. Prod. Res..

[57] Ishida Y., Hirota T., Sato S., Kamegai M., Kim S.-K., Park S.-Y., Koo D.-H., Shon M.-S., Kim G.-N., Park H.-R. (2015). Discriminative analysis of free and esterified gallic acids in acorn shells by thermochemolysis-gas chromatography/mass spectrometry in the presence of organic alkalis. J. Anal. Appl. Pyrolysis.

[58] Pasqualone A., Makhlouf F.Z., Barkat M., Difonzo G., Summo C., Squeo G., Caponio F. (2019). Effect of acorn flour on the physico-chemical and sensory properties of biscuits. Heliyon.

[59] Lv Y., Yu Z., Li C., Zhou J., Lv X., Chen J., Wei M., Liu J., Yu X., Wang C. (2022). Gelatin-based nanofiber membranes loaded with curcumin and borneol as a sustainable wound dressing. Int. J. Biol. Macromol..

[60] Saikaew R., Marsal P., Grenier B., Dubas S.T. (2018). Temperature controlled loading and release of curcumin in polyelectrolyte multilayers thin films. Mater. Lett..

[61] Ghasemi-Mobarakeh L., Werzer O., Keimel R., Kolahreez D., Hadley P., Coclite A.M. (2019). Manipulating drug release from tridimensional porous substrates coated by initiated chemical vapor deposition. J. Appl. Polym. Sci..

[62] Emanet M., Okuda M., Sen O., Lavarello C., Petretto A., Takeoka S., Ciofani G. (2022). Sumac (*Rhus coriaria*) Extract-Loaded Polymeric Nanosheets Efficiently Protect Human Dermal Fibroblasts from Oxidative Stress ?. ACS Appl. Bio Mater..

[63] Mirian M., Behrooeian M., Ghanadian M., Dana N., Sadeghi-Aliabadi H. (2015). Cytotoxicity and antiangiogenic effects of *Rhus coriaria*, *Pistacia vera* and *Pistacia khinjuk* oleoresin methanol extracts. Res. Pharm. Sci..

[64] Abdallah S., Abu-Reidah I., Mousa A., Abdel-Latif T. (2019). *Rhus coriaria* (sumac) extract reduces migration capacity of uterus cervix cancer cells. Rev. Bras. Farmacogn. -Braz. J. Pharmacogn..

[65] Batiha G.E.-S., Beshbishy A.M., Adeyemi O.S., Nadwa E.H., Rashwan E.K.M., Alkazmi L.M., Elkelish A.A., Igarashi I. (2020). Phytochemical Screening and Antiprotozoal Effects of the Methanolic *Berberis vulgaris* and Acetonic *Rhus coriaria* Extracts. Molecules.

[66] Bostanci N.S., Buyuksungur S., Hasirci N., Tezcaner A. (2022). pH responsive release of curcumin from photocrosslinked pectin/gelatin hydrogel wound dressings. Biomater. Adv..

[67] Farmacopeea Romana, ED. 101993_201802. https://archive.org/details/FARMACOPEEAROM.ED.101993_201802/page/n215/mode/2up.

[68] (2006). Piele. Analize Chimice. Determinarea Materiilor Volatile. https://magazin.asro.ro/Search?q=SR+EN+ISO+4684%3A2006.

[69] (1990). PH-Metrie. Scara de pH a Soluţiilor Apoase. https://magazin.asro.ro/ro/standard/21070.

[70] (1997). Calitatea Apei. Determinarea Conductivităţii Electrice. https://magazin.asro.ro/Search?q=EN+ISO+27883&ics=&l=&sw=1.

[71] Gmia Standard Methods for the Testing of Edible Gelatin. https://www.gelatin-gmia.com/.

[72] Rapa M., Zaharescu T., Stefan L.M., Gaidau C., Stanculescu I., Constantinescu R.R., Stanca M. (2022). Bioactivity and Thermal Stability of Collagen-Chitosan Containing Lemongrass Essential Oil for Potential Medical Applications. Polymers.

[73] (2009). Evaluarea Biologică a Dispozitivelor Medicale. Partea 5: Teste Pentru Citotoxicitate in Vitro. https://magazin.asro.ro/ro/standard/177544.

[74] (2019). Sterilization of Health Care Products—Microbiological Methods. https://www.iso.org/standard/70801.html.

